# Influenza A Virus (H3N8) in Dogs with Respiratory Disease, Florida

**DOI:** 10.3201/eid1406.071270

**Published:** 2008-06

**Authors:** Sunchai Payungporn, P. Cynda Crawford, Theodore S. Kouo, Li-mei Chen, Justine Pompey, William L. Castleman, Edward J. Dubovi, Jacqueline M. Katz, Ruben O. Donis

**Affiliations:** * Centers for Disease Control and Prevention, Atlanta, Georgia, USA; † University of Florida, Gainesville, Florida, USA; ‡ Cornell University, Ithaca, New York, USA

**Keywords:** Influenza type A virus, canine influenza, hemagglutinin gene sequence, molecular phylogeny, research

## Abstract

In 2004, canine influenza virus subtype H3N8 emerged in greyhounds in the United States. Subsequent serologic evidence indicated virus circulation in dog breeds other than greyhounds, but the virus had not been isolated from affected animals. In 2005, we conducted virologic investigation of 7 nongreyhound dogs that died from respiratory disease in Florida and isolated influenza subtype H3N8 virus. Antigenic and genetic analysis of A/canine/Jacksonville/2005 (H3N8) and A/canine/Miami/2005 (H3N8) found similarity to earlier isolates from greyhounds, which indicates that canine influenza viruses are not restricted to greyhounds. The hemagglutinin contained 5 conserved amino acid differences that distinguish canine from equine lineages. The antigenic homogeneity of the canine viruses suggests that measurable antigenic drift has not yet occurred. Continued surveillance and antigenic analyses should monitor possible emergence of antigenic variants of canine influenza virus.

## Abstract 2

Genetic and antigenic characterization established the uniqueness of this virus circulating in dogs.

*Influenza A viruses* (family *Orthomyxoviridae)* are known to cause acute respiratory disease in humans, horses, pigs, and domestic poultry (*1**,**2*). Influenza A virus subtype H3N8 has recently emerged as a respiratory pathogen in dogs, associated with outbreaks of acute respiratory disease in racing greyhounds (*3*). The disease is caused by a novel virus closely related to contemporary equine influenza A virus subtype H3N8. These viruses share >96% nucleotide sequence identity, which suggests direct transmission of the entire virus from horses to dogs without reassortment with other strains (*3*).

Canine influenza virus (CIV) was first identified in racing greyhounds in Florida in January 2004 and was later associated with respiratory disease outbreaks in racing greyhounds in 9 states from 2004 through 2006 (*3**,**4*). Most affected greyhounds had clinical signs associated with virus infection of the upper respiratory tract—cough for 10–30 days, nasal discharge, low-grade fever—followed by recovery. However, some dogs died peracutely with extensive hemorrhage in the lungs, mediastinum, and pleural cavity. Histologic examination showed tracheitis, bronchitis, bronchiolitis, and suppurative bronchopneumonia associated with extensive erosion of epithelial cells and infiltration with neutrophils. The isolation of 4 closely related influenza A subtype H3N8 viruses from dogs that died in different geographic locations over a 25-month period, together with substantial serologic evidence of widespread infection among racing greyhounds in 9 states, suggested sustained CIV circulation in this population by dog-to-dog transmission (*3**,**4*).

The first evidence of CIV infection in dogs other than greyhounds came from serologic testing of dogs with acute respiratory disease in shelters, boarding kennels, and veterinary clinics in Florida and New York in 2004 and 2005 (*3*). Since August 2005, a national syndromic serosurvey for canine influenza has been conducted on >5,000 samples collected from nongreyhound dogs with compatible clinical signs (Cornell University College of Veterinary Medicine, http://diaglab.vet.cornell.edu/issues/civ-stat.asp). As of April 2008, seropositive dogs have been identified in 25 states and the District of Columbia.

In April and May 2005, an outbreak of respiratory disease occurred in dogs housed in a shelter facility in northeastern Florida (*3*). The outbreak involved at least 58 dogs, ranging in age from 3 months to 9 years, and included purebred dogs as well as mixed breeds; 6 were euthanized. In May 2005, a respiratory disease outbreak occurred among ≈40 pet dogs at a veterinary clinic in southeastern Florida; 1 died. We performed molecular analyses on 2 influenza A subtype H3N8 viruses isolated from these 7 nongreyhound dogs that died and genetically and antigenically compared them with influenza (H3N8) viruses from racing greyhounds.

## Materials and Methods

### Specimen Collection

Postmortem examinations were performed on the 6 mixed-breed shelter dogs that died in April and May 2005 and on the 1 pet Yorkshire terrier that died in the veterinary clinic in May 2005. Tissues were fixed in 10% neutral buffered formalin and embedded in paraffin; 5-μm sections were stained with hematoxylin and eosin for histopathologic diagnosis. Unfixed tissues for virologic and molecular analyses were stored at –80^o^C.

### RNA Extraction

Frozen lung tissues from each dog were thawed and homogenized in lysis buffer containing β-mercaptoethanol by using a disposable tissue grinder (Kendall, Lifeline Medical Inc., Danbury, CT, USA). Total RNA was extracted by using a commercial kit (RNeasy Mini Kit, QIAGEN Inc., Valencia, CA, USA) according to manufacturer’s instructions and eluted in a final volume of 60 μL of buffer. Total RNA was also extracted from lung tissue collected from specific-pathogen–free dogs without respiratory disease.

### Real-Time Reverse Transcription–PCR

A single-step quantitative real-time reverse transcription–PCR (RT-PCR) was performed on total RNA extracted from the canine tissue samples by using the QuantiTect Probe RT-PCR Kit containing ROX as a passive reference dye (QIAGEN). Briefly, 2 primer-probe sets were used for detection of influenza A sequences in each sample (Table 1). One primer-probe set was selective for canine influenza subtype H3 gene sequences. The other primer-probe set targeted a highly conserved region of the matrix (M) gene of type A influenza virus. The sequence of the M probe contained 3 locked nucleic acids. For each real-time RT-PCR, 5 μL of total RNA was added to a reaction mixture containing 12.5 μL of 2× QuantiTect Probe RT-PCR Master Mix, 0.25 μL of QuantiTech RT Mix (both QIAGEN), forward and reverse primers (0.4 μmol/L final concentration for each), probe (0.1 μmol/L final concentration), and RNase-free water in a final volume of 25 μL. Real-time PCR for eukaryotic 18S rRNA was performed by using commercially available assay reagents (VIC/TAMRA; TaqMan, Applied Biosystems, Foster City, CA, USA), according to manufacturer’s instructions for detection of endogenous 18S rRNA, as an internal control for RNA extraction from the tissues.

**Table 1:** Primers and probes for identification of canine influenza virus (H3N8) in tissues by quantitative real-time reverse transcription–PCR*

Primer	Target	Sequence	Application
Ca-H3-F387	H3 (nt 387–406)	5′-tatgcatcgctccgatccat-3′	Forward primer for H3
Ca-H3-R487	H3 (nt 487–467)	5′-gctccacttcttccgttttga-3′	Reverse primer for H3
Ca-H3-P430	H3 (nt 430–459)	FAM-aattcacagcagagggattcacatggacag-BHQ1	TaqMan probe
FluA-M-F151	M (nt 151–174)	5′-catggartggctaaagacaagacc-3′	Forward primer for M
FluA-M-R276	M (nt 276–253)	5′-agggcattttggacaaakcgtcta-3′	Reverse primer for M
FluA-M-P218	M (nt 218–235)	FAM-acgcTcaccgTgcccAgt-BHQ1	TaqMan probe

*Conserved regions of the matrix (M) and hemagglutinin 3 (H3) genes of canine influenza virus were selected. The underlined r represents a mixture of a and g nucleotides at this position in the oligonucleotide; the underlined k represents mixtures of g or t; uppercase letters in the sequence for the M probe represent locked nucleic acids.

Quantitative one-step real-time RT-PCR was performed on the reaction mixtures in an Mx3000P QPCR System (Stratagene, La Jolla, CA, USA). Cycling conditions were a reverse transcription step at 50°C for 30 min, an initial denaturation step at 95°C for 15 min to activate the HotStarTaq DNA Polymerase (QIAGEN), and amplification for 40 cycles. Each amplification cycle included denaturation at 94°C for 15 s, followed by annealing/extension at 60°C for 1 min. The FAM (emission wavelength 516 nm) and VIC (emission wavelength 555 nm) fluorescent signals were recorded at the end of each cycle. The threshold cycle (Ct) was determined by setting the threshold fluorescence at 1,000 for each individual experiment. The Mx3000P version 2.0 software program (Stratagene) was used for data acquisition and analysis. The results were normalized by calculating H3 Ct or M Ct to 18S rRNA Ct ratios for each sample. Samples were considered positive for influenza A virus when the normalized H3 or M Ct ratio was 3 U smaller than the normalized Ct ratio for lung tissues from dogs without respiratory disease. The positive control was amplified RNA that had been extracted from A/canine/Florida/242/2003 (H3N8) virus grown in MDCK cells (*3*).

### Virus Isolation

#### Inoculation of Cell Culture

Frozen lung tissues from each of the 7 dogs were thawed and homogenized in 10 volumes of Dulbecco modified Eagle medium (DMEM) supplemented with 0.5% bovine serum albumin and gentamicin and ciprofloxacin. Solid debris was removed by centrifugation, and supernatants were inoculated onto MDCK cells cultured in DMEM supplemented with 1 μg/mL TPCK (L-1-tosylamido-2-phenylethyl chloromethyl ketone)–treated trypsin (Sigma-Aldrich Corp., St. Louis, MO, USA), 0.35% bovine serum albumin, and antimicrobial drugs. Cells were grown in 25-cm^2^ flasks at 37°C in a humidified atmosphere containing 5% CO_2_. The cultures were observed daily for morphologic changes and harvested 3 days after inoculation. The harvested cultures were clarified by centrifugation, and the supernatants were inoculated onto fresh MDCK cells as described for the initial inoculation; up to 3 additional passages were performed for samples that did not show evidence of influenza virus by hemagglutination or RT-PCR. Hemagglutination activity in the clarified supernatants was determined by using 0.5% turkey red blood cells as described (*5*,*6*). RT-PCR was performed as described below.

#### Inoculation of Embryonated Chicken Eggs

Frozen lung tissues were homogenized as described above for inoculation of MDCK cells by using phosphate-buffered saline instead of DMEM, and a volume of 0.2 mL was inoculated into the allantoic sac of 10-day old embryonated chicken eggs. After 48 h of incubation at 35°C, the eggs were chilled at 4°C overnight before the allantoic fluid was harvested. Hemagglutination activity in the clarified allantoic fluid supernatants was determined by using 0.5% turkey red blood cells as described (*5**,**6*). RT-PCR was performed as described below. Harvested allantoic fluid samples lacking evidence of influenza virus were reinoculated up to 2 more times in embryonated eggs and evaluated as described.

### RT-PCR, Nucleotide Sequencing, and Phylogenetic Analyses

Virus RNA was extracted from MDCK culture supernatant or allantoic fluid by using the QIAamp Viral RNA Mini Kit (QIAGEN) according to manufacturer’s instructions. The virus RNA was reverse transcribed to cDNA by using the QIAGEN OneStep RT-PCR Kit according to manufacturer’s instructions. PCR amplification of the coding region of the 8 influenza virus genes in the cDNA was performed as described (*7*) by using universal gene-specific primer sets (*3*). The resulting DNA amplicons were used as templates for automated sequencing in the ABI PRISM 3100 automated DNA sequencer by using cycle sequencing dye terminator chemistry (Applied Biosystems). Nucleotide sequences were analyzed by using the Lasergene 6 Package (DNASTAR, Inc., Madison, WI, USA). The PHYLIP Version 3.5 software program was used to estimate phylogenies and calculate bootstrap values from the nucleotide sequences (*8*). Phylogenetic trees were compared with those generated by neighbor-joining analysis with the Tamura-Nei model implemented in the MEGA3 program (*9*) and confirmed by the PAUP 4.0 Beta program (Sinauer Associates, Inc., Sunderland, MA, USA). The complete genome sequences from the 2 new reported CIV isolates (A/canine/Miami/2005 and A/canine/Jacksonville/2005) were deposited in GenBank under accession nos. EU402407–EU402408 and EU534193–EU534204.

### Hemagglutination Inhibition Assay

Convalescent-phase immune serum samples were obtained from horses and dogs naturally infected with influenza A virus (H3N8) in 2005. Antiserum from ferrets infected with A/canine/Florida/43/2004 (H3N8) was prepared as described (*3*). All serum samples were incubated with receptor-destroying enzyme (DENKA SEIKEN Co., Ltd., Tokyo, Japan) (1 part serum: 3 parts receptor-destroying enzyme) for 16 h at 37°C before heat inactivation for 30 min at 56°C. Influenza A/canine/Jacksonville/2005 virus (H3N8) was grown in MDCK cells for 72 h at 37°C in 5% CO_2_. Virus culture supernatants were harvested, clarified by centrifugation, and stored at –80°C. All other canine and equine viruses used in the hemagglutination inhibition (HI) assay were grown in 10-day old embryonated chicken eggs from which allantoic fluid was collected after 72 h and stored at –80°C. The HI assay was performed as described (*6*). On the basis of assay results from serum of uninfected specific-pathogen–free dogs with HI titers <4, HI titers >32 were considered as evidence of previous exposure to CIV.

## Results

### Clinical Findings

Among the 58 affected shelter dogs, the most common clinical signs were low-grade fever, purulent nasal discharge, and cough for 10–21 days. Paired acute- and convalescent-phase serum samples were collected from 5 dogs and tested for CIV-specific antibodies by using the HI assay. All the dogs seroconverted to A/canine/Florida/43/2004 (H3N8) and had an increase in the geometric mean antibody titer (GMT) from 37 in the acute phase to 626 in the convalescent phase. Single serum samples were collected from another 18 dogs that had had clinical disease for at least 7 days, and 17 (94%) were seropositive for A/canine/Florida/43/2004 (H3N8); HI antibody titers ranged from 32 to 2,048 and GMT was 533. Pneumonia developed in at least 10 dogs, of which 6 were euthanized and submitted for postmortem examination. These 6 mixed-breed dogs were 3 males and 3 females ranging in age from 4 months to 3 years. The duration of clinical signs at the time of euthanasia was 2–10 days.

Among the ≈40 affected pet dogs at the veterinary clinic, the most common clinical signs were low-grade fever, purulent nasal discharge, and cough for 10–30 days. Paired acute- and convalescent-phase serum samples were collected from 19 dogs and tested for CIV-specific antibodies by using the HI assay. Of these, 11 (58%) dogs seroconverted to A/canine/Florida/43/2004 (H3N8), and the GMT increased from 9 in the acute phase to 329 in the convalescent phase. Single serum samples were collected from another 9 dogs that had had clinical disease for at least 7 days, and 6 (67%) were seropositive for A/canine/Florida/43/2004 (H3N8); HI antibody titers ranged from 64 to 512 and GMT was 228. Pneumonia developed in 3 dogs; 1, a 9-year-old male Yorkshire terrier, died 3 days after onset of clinical signs and was submitted for postmortem examination.

Postmortem examinations showed that all 7 dogs had tracheitis and bronchitis with inflammatory changes that involved submucosal glands. Tracheitis and bronchitis were characterized by surface and glandular epithelial necrosis and hyperplasia with infiltration by lymphocytes, neutrophils, and macrophages. Suppurative bronchopneumonia was found in 2 of the shelter dogs and the pet dog; histologically identified bacteria in the lesions indicated a bacterial contribution to the pneumonic lesions. None of the dogs had intrathoracic or pulmonary hemorrhage.

### Laboratory Diagnosis

Lung tissues from the 7 dogs were analyzed by quantitative real-time RT-PCR assays that detect the M gene of influenza A and the H3 gene of CIV. The M and H3 genes were amplified from the lungs of all 7 dogs, which confirmed the presence of CIV (Table 2). Lung tissue from the specific-pathogen–free dogs did not show evidence of amplification of influenza virus genes. MDCK cultures were inoculated with these lung homogenates to identify viable influenza virus. Pathologic cell damage was noted in the MDCK cell monolayer on the third passage of the lung homogenate from 1 of the shelter dogs that died after 3 days of pneumonia. Influenza A virus (later identified as subtype H3N8) was recovered from the supernatant, and the isolate was named A/canine/Jacksonville/2005. After 2 passages in embryonated chicken eggs, influenza A virus (later identified as subtype H3N8) was recovered from the lung homogenate of the pet dog that also died of pneumonia 3 days after onset. This virus was named A/canine/Miami/2005. These isolates provided virologic evidence of CIV infection in nongreyhound dogs.

**Table 2:** Quantitative real-time reverse transcription–PCR and virus isolation from tissue specimens*

Dog	Location	Duration of clinical disease, d	Real-time reverse transcription–PCR					
			18S rRNA (Ct)	M (Ct)	M:rRNA	H3 (Ct)	H3:rRNA	Virus isolation
1079	Shelter	2	26.18	29.81	1.14	28.84	1.10	NVD
1078	Shelter	3	26.82	30.37	1.13	29.71	1.11	MDCK cells 3rd passage
1080	Shelter	6	24.17	38.87	1.61	38.23	1.58	NVD
319	Shelter	6	24.94	33.87	1.36	32.23	1.29	NVD
318	Shelter	9	23.54	33.89	1.44	32.97	1.40	NVD
320	Shelter	10	23.91	39.44	1.65	37.09	1.55	NVD
374	Veterinary clinic	3	22.89	24.05	1.05	22.65	0.99	Egg 2nd passage
A/canine/Florida/242/2003			29.37	28.15	0.96	27.36	0.93	NA
Normal dog lung			20.78	>40	>1.92	>40	>1.92	NA

*Ct, cycle threshold; NVD, no virus detected; NA, not applicable; M:rRNA and H3:rRNA ratios were calculated by dividing the matrix (M) or hemagglutinin 3 (H3) Ct by the 18S rRNA Ct.

### Genetic Analyses of the Canine Influenza A (H3N8) Isolates

Sequence analyses of A/canine/Jacksonville/2005 and A/canine/Miami/2005 showed that their H3 gene nucleotide sequences were 98% identical to those of the A/canine/Florida/242/2003, A/canine/Florida/43/2004, A/canine/Texas/2004, and A/canine/Iowa/2005 isolates recovered from the lungs of racing greyhounds that died of pneumonia during influenza outbreaks in 2004 and 2005 (*3**,**4*). Phylogenetic comparisons of the H3 genes showed that the A/canine/Jacksonville/2005 and A/canine/Miami/2005 viruses from nongreyhound dogs clustered with the greyhound isolates and contemporary equine isolates, forming a distinct group from the older equine viruses isolated in the early 1990s (Figure, **panel A**). Furthermore, the A/canine/Jacksonville/2005, A/canine/Miami/2005, and A/canine/Iowa/2005 isolates were more closely related to A/canine/Texas/2004 than to either A/canine/Florida/43/2004 or A/canine/Florida/242/2003. The H3 genes from the 2005 isolates formed a subgroup that appeared to branch off from the earlier 2003 and 2004 canine viruses with nucleotide differences at 10 sites. Most nucleotide changes are silent, as can be appreciated by the shorter branch lengths in the phylogenetic tree constructed from deduced amino acid sequence data (Figure, **panel B**).

**Figure:**
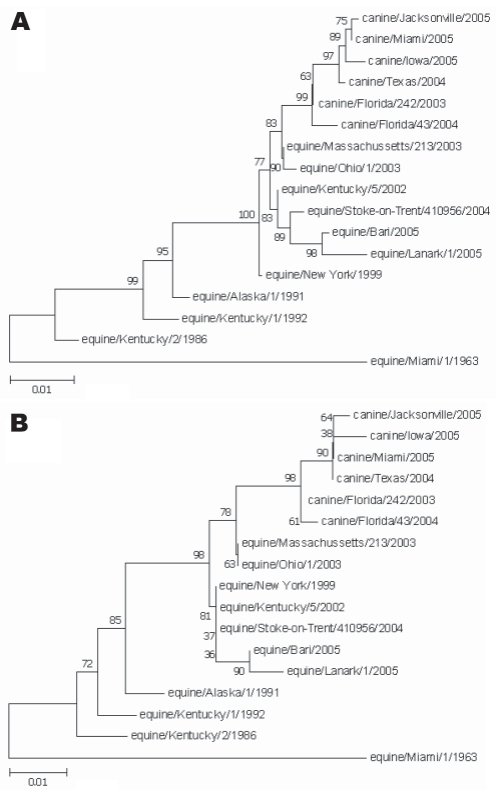
Phylogenetic relationships among the hemagglutinin 3 (H3) genes. A) Nucleotide tree of the canine influenza virus H3 genes with contemporary and older equine H3 genes. B) Amino acid tree of the canine influenza virus H3 protein with contemporary and older equine H3 proteins. Bootstrap analysis values >80% are shown. Scale bar indicates nucleotide or amino acid substitutions per site.

### Amino Acid Sequence of the CIV H3 Hemagglutinin

To identify changes with possible functional significance with regard to antigenicity or receptor binding, we compared the deduced amino acid sequences of the H3 hemagglutinins from the 6 available CIV isolates. All 6 canine isolates had 5 conserved amino acid substitutions that differentiated them from contemporary equine influenza viruses (Table 3). These conserved substitutions (N54K, N83S, W222L, I328T, and N483T) can be considered as a signature of the circulating CIV H3 hemagglutinin. Phylogenetic comparisons of the mature H3 protein showed that the A/canine/Jacksonville/2005, A/canine/Miami/2005, and A/canine/Iowa/2005 viruses formed a subgroup with the A/canine/Texas/2004 isolate (Figure, **panel B**). Three amino acid changes (L118V, K261N, and G479E) differentiated this subgroup from the earlier A/canine/Florida/43/2004 and A/canine/Florida/242/2003 isolates (Table 3). Finally, A/canine/Jacksonville/2005 differed from A/canine/Miami/2005 at a single amino acid, S107P.

**Table 3:** Amino acid differences among the hemagglutinin proteins of canine and contemporary equine influenza viruses* http://www.cdc.gov/Eid/content/14/6/902-T3.htm

Virus	Amino acid positions in mature HA																	
	7†	29	**54**	78	79	**83**	92	107	118	159	218	**222**	261	**328**	479	**483**	492	541
A/equine/Kentucky/5/2002	G	I	N	V	F	N	S	S	L	N	G	W	K	I	G	N	R	K
A/equine/Massachusetts/213/2003	**.**	**.**	**.**	A	**.**	**.**	**.**	**.**	**.**	S	**.**	**.**	**.**	**.**	**.**	**.**	**.**	**.**
A/equine/Ohio/1/2003	**.**	**.**	**.**	A	**.**	**.**	**.**	**.**	**.**	S	**.**	**.**	**.**	**.**	**.**	**.**	**.**	**.**
A/equine/Bari/2005	D	**.**	**.**	**.**	**.**	**.**	**.**	**.**	**.**	**.**	**.**	**.**	**.**	**.**	**.**	**.**	**.**	**.**
A/canine/Florida/242/2003	D	**.**	**K**	A	**.**	**S**	**.**	**.**	**.**	S	**.**	**L**	**.**	**T**	**.**	**T**	**.**	**.**
A/canine/Florida/43/2004	**.**	M	**K**	A	**.**	**S**	N	**.**	**.**	S	**.**	**L**	**.**	**T**	**.**	**T**	**.**	R
A/canine/Texas/1/2004	**.**	M	**K**	A	**.**	**S**	**.**	**.**	**V**	S	**.**	**L**	**N**	**T**	**E**	**T**	**.**	**.**
A/canine/Iowa/2005	**.**	M	**K**	A	**L**	**S**	**.**	**.**	**V**	S	**E**	**L**	**N**	**T**	**E**	**T**	**.**	**.**
A/canine/Miami/2005	**.**	M	**K**	A	**.**	**S**	**.**	**.**	**V**	S	**.**	**L**	**N**	**T**	**E**	**T**	K	**.**
A/canine/Jacksonville/2005	**.**	M	**K**	A	**.**	**S**	**.**	P	**V**	S	**.**	**L**	**N**	**T**	**E**	**T**	K	**.**

*Dots represent the same amino acids as in A/equine/Kentucky/5/2002 hemagglutinin (HA). Conserved residues in the canine HA that differentiate them from the equine HA are in **boldface** within blue-shaded boxes. Residues that differentiate the A/canine/Texas/2004 clade from A/canine/Florida/242/2003 and A/canine/Florida/43/2004 are in **boldface** within gray-shaded boxes. †Only variable positions are shown; numbering is for mature HAO.

### Antigenic Analyses of the Canine Influenza A (H3N8) Isolates

HI tests were performed by using an antigen panel of previously circulating and contemporary equine influenza viruses and the available CIVs as well as convalescent-phase immune serum from horses and dogs naturally infected with influenza virus (H3N8) in 2005 (Table 4). An antiserum from ferrets infected with A/canine/Florida/43/2004 was also included in the analyses. HI antibody titers in equine serum were 2-fold to 16-fold higher with contemporary equine viruses (1999–2003) than with older isolates (1963–1992). The heterologous titers of equine serum to canine viruses were generally similar to the homologous values for the contemporary equine viruses. The canine serum failed to substantially inhibit hemagglutination by the older equine influenza viruses (1963–1992), but the antibody titers to contemporary equine isolates (1999–2003) and the canine isolates were comparable. Similar results were observed for serum from ferrets infected with CIV. These patterns of inhibition demonstrated the antigenic similarity between the CIVs and contemporary equine influenza viruses and were consistent with the phylogenetic analyses. The antibody titers to the A/canine/Miami/2005 isolate in equine, canine, and ferret serum were similar to those for the 2003 and 2004 canine isolates, which indicates that the amino acid substitutions in the isolates did not result in measurable antigenic drift. The antibody titers to the A/canine/Jacksonville/2005 isolate were, in general, 2- to 4-fold lower than those to A/canine/Florida/43/2004 and other canine viruses, which suggests a potential antigenic difference.

**Table 4:** Hemagglutination inhibition antibody titers to older and contemporary equine viruses and canine influenza viruses*

Virus	Serum sample no.								
	Equine†				Canine‡				Ferret§
	65694	73147	84376		13	25	27		A/canine/Florida/43/2004
A/equine/Miami/63	40	40	160		<10	<10	10		16
A/equine/Kentucky/86	40	40	160		10	40	40		32
A/equine/Kentucky/92	40	20	80		<10	<10	10		32
A/equine/NewYork/99	320	40	320		40	160	40		128
A/equine/Kentucky/05/2002	320	40	320		40	160	160		256
A/equine/Massachussetts/213/2003	640	80	320		40	160	160		512
A/equine/Ohio/01/2003	640	80	320		80	320	160		512
A/canine/Florida/242/2003	160	40	320		40	160	160		512
A/canine/Texas/2004	160	40	320		40	160	160		512
A/canine/Florida/43/2004	160	40	320		40	160	80		512
A/canine/Miami/2005	320	40	320		40	160	80		256
A/canine/Jacksonville/2005	40	10	80		20	40	40		128

*The antibody titer is the reciprocal of the highest serum dilution that inhibited hemagglutination by the virus. †Serum from horses infected with equine influenza virus in 2005. ‡Serum from dogs infected with canine influenza virus in 2005. §Serum from ferrets infected with A/canine/Florida/43/2004 (H3N8).

## Discussion

The genetic and phylogenetic analyses of influenza A subtype H3N8 viruses recovered from racing greyhounds affected by respiratory disease outbreaks and fatal pneumonia in 2003 and 2004 have been described (*3*). These greyhound influenza viruses were most homologous to contemporary equine influenza A subtype H3N8 viruses isolated from horses in 2002 and 2003. Although serologic evidence of influenza virus infection in nongreyhound dogs was reported (*3*), whether these infections were caused by the same virus that infected the greyhound dogs is not clear. The influenza subtype H3N8 viruses that we describe in this report came from nongreyhound dogs involved in fatal canine influenza outbreaks independent of any known outbreaks in greyhounds.

Because viral hemagglutinin is a critical determinant of the host specificity of influenza virus (*10*), we compared the nucleic acid and deduced amino acid sequences for the canine and equine H3 to identify residues that may be associated with efficient replication in different species or dog breeds. The 5 conserved amino acid substitutions in all the canine viruses differentiated them from contemporary equine viruses. The substitution of lysine for asparagine at position 54 maintains the positive charge of the residue and is of unknown functional significance. Position 83 located within antigenic site E of human H3 has been implicated in antigenic drift (*11*). The substitution of serine for asparagine at position 83 in canine H3 maintains the polar nature of the residue, but the functional significance in the evolution of canine influenza is not immediately apparent. The substitution of leucine for tryptophane at position 222 represents a nonconservative change adjacent to the sialic acid–binding pocket, which suggests a potential modulator function in adaptation of equine influenza virus to canine sialic acid receptors. This leucine substitution has also been reported in avian influenza subtype H4 infection of pigs (*12*) and subtype H9 infection of humans (*13*). The strictly conserved isoleucine at position 328 near the cleavage site of the equine H3 hemagglutinin has been replaced by threonine in all the canine isolates, which suggests the potential importance of threonine for recognition of the hemagglutinin cleavage site by canine proteases. The replacement of asparagine with threonine at position 483 results in the loss of a glycosylation site in the hemagglutinin 2 (HA2) subunit. This glycosylation site is conserved in all other hemagglutinin subtypes (*14*).

The phylogenetic tree of the canine and equine influenza H3 genes shows that the canine and equine lineages have diverged considerably. The hemagglutinin sequences from the 2004 and 2005 equine influenza isolates belong to the Florida sublineage and do not have the mutations found in the canine strains. The H3 genes from the 2 Florida 2005 canine isolates formed a clade with high bootstrap support that included A/canine/Texas/2004 and A/canine/Iowa/2005. Three amino acid substitutions in H3—L118V, K261N, and G479E—differentiated this group from the earlier isolates. Positions 118 and 261 are in the hemagglutinin 1 (HA1) subunit of canine H3; position 479 is in the HA2 subunit. The HA1 subunit of human H3 contains the antibody-binding sites where amino acid substitutions occur at high frequency, presumably the result of escape from humoral immune responses (*11**,**15**,**16*). Although no evidence supports high variability at positions 118 and 261, neighboring positions 121 (antigenic site D) and 262 (antigenic site E) are sites of frequent positive selection in human H3 genes (*11*). The 2005 isolates from nongreyhound dogs differed from each other by substitution of proline for serine at position 107 in the HA1 subunit of A/canine/Jacksonville/2005. Serine is conserved at position 107 in all other equine and canine isolates except for A/equine/Jilin/1/89, which has a threonine substitution (*17*). A/canine/Jacksonville/2005 has potentially significant antigenic variation from the other canine isolates, which may be partially related to the proline substitution at position 107. However, no serologic evidence indicates that residue 107 modulates the antigenicity of human, porcine, or equine H3 hemagglutinins.

The HI results showed antigenic similarity between the canine and contemporary equine influenza viruses and were in agreement with the phylogenetic clustering of canine hemagglutinin sequences near the equine counterparts. The amino acid and antigenic differences between canine and equine hemagglutinin illustrate the ongoing process of divergent evolution of the canine viruses from the ancestral equine viruses. These differences also support the hypothesis that CIV is a separate virus lineage, which is efficiently maintained in the dog population by horizontal dog-to-dog transmission.

With the introduction and sustained transmission of influenza virus (H3N8) in dogs, the H3 hemagglutinin became the most broadly distributed subtype in mammalian species, including humans, swine, horses, and dogs. Continued virologic and serologic surveillance will be important for monitoring the evolution of CIV and its health effects in dogs as well as the possible transmission to other species, including humans.
